# Spherical spiky ZnO/Au nanostructures for efficient photoelectrochemical water splitting

**DOI:** 10.55730/1300-0527.3718

**Published:** 2024-11-02

**Authors:** Kouroush SALİMİ

**Affiliations:** Department of Chemical Engineering, Faculty of Engineering and Natural Sciences, Ankara Yildirim Beyazit University, Ankara, Turkiye

**Keywords:** Spiky ZnO, gold nanoparticles, polydopamine, H_2_ generation, water splitting

## Abstract

Spiky zinc oxide (ZnO)/Au nanostructures with spherical shape-defined morphologies were synthesized using polydopamine as a starting template for photoelectrochemical H_2_ production under visible light-emitting diode (LED) illumination. This low-temperature processing technique not only facilitated the fabrication of ideal plasmon-sensitive heterostructures, but also enhanced the electron mobility of the photoanodes, reaching 5.7 mA/cm^2^ [at 1.0 V vs. the reversible hydrogen electrode (RHE)] under visible LED illumination (30 mW/cm^2^). This notable value was 28 times greater than that observed under dark conditions, primarily attributed to the close Schottky contact between the Au and ZnO spikes. The highest applied bias photon-to-current efficiency value (6.0%, at 0.81 V vs. the RHE) and good incident photon-to-current conversion efficiency, particularly in the visible region, coupled with a significant decrease in photoluminescence intensity, was achieved for the ZnO/Au photoanodes, owing to the improved light harvesting capability and effective electron-hole separation, resulting in the injection of hot electrons from Au to the conduction band of the spiky ZnO. This unique synthesis technique revealed a new generation of visible-light responsive plasmonic heterostructures with regular morphologies for efficient conversion of solar to H_2_ fuels and energy storage applications.

## 1. Introduction

Photoelectrochemical (PEC) water splitting represents a more environmentally friendly method for hydrogen generation compared to other detrimental technologies [1]. In this approach, electrodes coated with specific semiconductors facilitate the production of hydrogen energy [1]. Materials with superior visible light absorption, enhanced charge carrier separation, and optimal bandgap positioning are crucial for the development of high-quality, cost-effective, stable, and photo corrosion-resistant photoelectrodes for this application [1–3]. Despite significant advancements, challenges such as low-temperature processing, cost effectiveness, and long-term stability remain focus areas. The performance of the PEC device is significantly influenced by the electron transfer layer (ETL), the hole transport layer (HTL), and counter electrode. In recent years, inorganic semiconductors with hierarchical structures, such as TiO_2_, ZnO, WO_3_, ZrO_2_, and BiVO_4_, among others, have emerged as promising ETL materials for PEC applications due to their unique properties [1]. Among these materials, zinc oxide (ZnO) is widely used as an ETL in PEC water splitting due to its high electron mobility (210 cm^2^ V^−1^ s^−1^) [4]. However, its wide band gap (approximately 3.37 eV) limits the photocatalytic efficacy to the ultra violet (UV) region of the solar spectrum, which represents only 5% of the incident light, resulting in low utilization of visible light [5,6]. Researchers have made various efforts to address these challenges, leading to improved ZnO photocatalytic performance through band gap reduction and enhanced visible light photoresponsivity [5,6].

Recently, the incorporation of plasmonic nanoparticles (NPs) and their synergy with inorganic semiconductors have led to advantages such as light harvesting, enhancing the efficiency of photoanodes across a broad wavelength range while facilitating charge separation and collection [4,7,8]. Furthermore, maximal oscillation amplitude can be achieved at specific wavelengths by manipulating the size, shape, morphology, and crystalline structure of the plasmonic NPs [9]. Consequently, the distinct optoelectronic effects of plasmonic nanostructures can be enhanced by adjusting the localized surface plasmon resonance (LSPR) wavelength, extinction cross-section, and incident light energy [4,9]. Abouelela et al. [10] demonstrated the use of Ag NPs-deposited ZnO nanopagodas for highly efficient PEC water splitting due to increased light harvesting. Güler et al. [5] produced ZnO/Au nanorods using photoreduction and annealing techniques. They achieved a significant increase in photocurrent generation (0.009 mA/cm^2^ at 1.1 V vs. the normal hydrogen electrode) due to the photoresponse of Au NPs, particularly in the visible band. Machin et al. [11] constructed biomimetic Au@ZnO-graphene composite catalysts that effectively harvest light under 400 nm irradiation for PEC hydrogen production. In another study, Wu et al. [6] developed matchlike ZnO/Au plasmonic heterostructures that exhibited the highest solar-to-hydrogen conversion efficiency (0.48%) when compared to a bare ZnO nanorod array. Recently, Au NPs were integrated into ZnO nanowires for UV visible (UV-vis) light-responsive hydrogen synthesis, achieving the maximum amount of hydrogen at 853 μmol/hg [12]. Despite these significant efforts, no research has been undertaken on the PEC water splitting of well-defined, low-temperature processed spiky ZnO/Au photoanodes with regular spherical morphologies under energy-efficient visible white light-emitting diode (LED) irradiation until now.

Herein, a systematic seed-mediated growth approach was applied to construct spiky ZnO/Au nanostructures for PEC water-splitting applications. The physicochemical characteristics and PEC properties of the newly produced nanostructures were examined. The improved photocatalytic activity was achieved due to the intimate contact between Au NPs and ZnO spikes, which created optimal plasmon-sensitive photoanodes and excellent absorption of visible light under white LED illumination.

## 2. Materials and methods

### 2.1. Chemicals

Dopamine hydrochloride (DOP-HCl), chloroauric acid (HAuCl_4_), hydrochloric acid (HCl), zinc acetate dihydrate (Zn(CH_3_COO)_2_·2H_2_O), zinc nitrate hexahydrate (Zn(NO_3_)_2_·6H_2_O), ammonia, Tris-HCL, sodium hydroxide (NaOH), potassium hydroxide (KOH), sodium sulfate (Na_2_SO_4_, anhydrous, ≥99.0 %), hexamethylenetetramine (HMT), and Nafion (Sigma-Aldrich Chemical Co., St. Louis, MO, USA), and absolute ethanol and sodium borohydride (Merck KGaA, Darmstadt, Germany) were purchased commercially. Ultrapure deionized water (18 MΩ) from a Millipore Direct-Q3 UV system (Merck Millipore, Burlington, MA, USA) was used in all the experiments.

### 2.2. Synthesis of the spherical spiky ZnO/Au nanostructures

Polydopamine (PDA) NPs with a narrow particle size distribution were synthesized using an in situ polymerization technique [13,14]. For this purpose, 43 mg of dopamine hydrochloride was dissolved in a solvent mixture containing water (12.5 mL) and ethanol (5.0 mL) while vigorously stirring for 10 min. The reaction mixture was then immediately injected with 0.35 mL of ammonia solution, and the stirring process was carried out at room temperature for 30 min. The reaction was subsequently stopped by centrifugation and decantation (at 14,000 rpm for 10 min). The resultant PDA NPs were rinsed three times with distilled water to eliminate any excess dopamine molecules that did not participate in the process.

The spherical ZnO/Au nanorods were produced as follows:

In the first step: ZnO seed NPs were synthesized using a specified recipe [15], in which 0.40 g of zinc acetate dihydrate was dissolved in 20 mL of methanol and slowly stirred for 30 min (solution A). Next, 0.30 g of KOH was dissolved in 10 mL of methanol and sonicated for 30 min (solution B). Solutions A and B were then combined and sonicated at 60 °C for 30 min. The reaction medium was cooled to room temperature and the resultant ZnO cores were collected from the reaction media using centrifugation-decantation processes (at 5000 rpm) and washed with methanol and water three times.

In the second step, PDA particles (1.0 mg) were sonicated in a 5 mL Tris-buffer solution (pH 8.5) at room temperature for 10 min. Then, ZnO seed NPs (1.0 mg) were added to the mixture and sonicated at room temperature for 2 min. The ZnO seed PDA colloid particles were extracted from the reaction media via centrifugation (at 4500 rpm) and rinsed three times with water. Finally, the ZnO seed PDA colloid particles were redispersed in deionized water for further usage.

In the third step, a mixture containing zinc nitrate hexahydrate (40 mM) and hexamethylenetetramine (HMT, 40 mM) was employed to induce the formation of ZnO spikes on the PDA. Then, the ZnO seed PDA colloid particles (1.0 mg) were introduced into the mixture and stirred at room temperature for 10 min to facilitate the formation of ZnO spikes on the PDA surface. The resulting mixture was then transferred to a Teflon-coated stainless steel reactor and subjected to a hydrothermal process at 90 °C for 2 h to generate ZnO nanorods. The resulting spherical shape-defined spiky ZnO nanostructures were promptly centrifuged from the reaction medium to eliminate any unwanted ZnO structures, and then washed five times with distilled water, twice with ethanol, and three times with deionized water, respectively.

In the last step, colloidal Au NPs were synthesized using the method of Martin [16]. For this purpose, a stock solution (stock A) of 50 mM HAuCl_4_ and 50 mM HCl was prepared and stored at 7 °C in the dark. A second stock solution (stock B), containing 50 mM sodium borohydride and 50 mM NaOH, was also produced and stored at 7 °C. To synthesize the Au NPs, 200 μL of stock A and 20 mL of distilled water were mixed at room temperature. Next, 600 μL of stock B solution was quickly added to the prepared solution and stirred for 5 min. The color of the final solution changed from light yellow to red. For the production of the ZnO/Au, 1.0 mg of spiky ZnO was added to a 10 mL colloidal solution of Au NPs, and the mixture was stirred at room temperature for 6 h. The synthesized ZnO/Au composites were designated as ZnO/Au_x_, where x represents the loading amount of Au nanoparticles by weight (x = 15 wt%). Finally, the plasmonic nanostructures were washed three times with distilled water and centrifuged-decanted (at 4500 rpm) to remove residues from the reaction medium.

### 2.3. Characterization of the plasmonic spiky ZnO/Au nanostructures

The morphology and elemental mapping of the plasmonic ZnO/Au nanorods were investigated using a scanning electron microscope (Hitachi SU5000, Hitachi Ltd., Chiyoda-ku, Tokyo, Japan) equipped with energy-dispersive X-ray spectrometry (EDX). X-ray diffraction (XRD) (Rigaku Miniflex 600; Rigaku Corporation, Tokyo, Japan) was utilized to examine the crystalline structure of nanostructures using Cu Kα1/2 radiation. The surface composition was determined using X-ray photoelectron spectroscopy (XPS) using a Thermo-K-Alpha-Monochromated Spectrometer (Thermo Fisher Scientific Inc., Waltham, MA, USA). UV-vis measurements were performed with a Shimadzu-UV 1601 spectrophotometer (Shimadzu Corp., Kyoto, Japan) in the range of 200–800 nm.

### 2.4. PEC H_2_ generation

PEC examination of the photocatalysts was performed using the CorrTest Electrochemical Workstation Potentiostat/Galvanostat equipment (Wuhan Corrtest Instruments Co., Ltd., Wuhan, China). In a three-electrode setup, Ag/AgCl and Pt wires served as the reference and counter electrodes, respectively. Measurements were carried out at room temperature in a 0.1 M Na_2_SO_4_ solution (pH: 7). A visible light source was designed using 96 surface-mounted device (SMD) LEDs with a 5760 SMD package size. The light intensity at the base of the chamber was 30 W/cm^2^, measured with a Solar Power Meter (Mastech SM206; Shenzhen Mastech Industrial Co., Ltd., Shenzhen, Guangdong, China). Photocatalyst-modified fluorine-doped tin oxide (FTO) glass served as a working electrode. First, the FTO was cleaned in an ultrasonic bath with a 50:50 (v/v) mixture of ethanol and water. The 0.2 cm^2^ surface area of the FTO glass was then modified by drop-coating with the photocatalyst dispersion in a mixture of water/ethanol (25:25 volumetric ratio) containing Nafion (5 wt%). The drop-casted samples were dried on a hot plate (100 °C) for 10 min under atmospheric conditions.

## 3. Results and discussion

**Figure 1:**
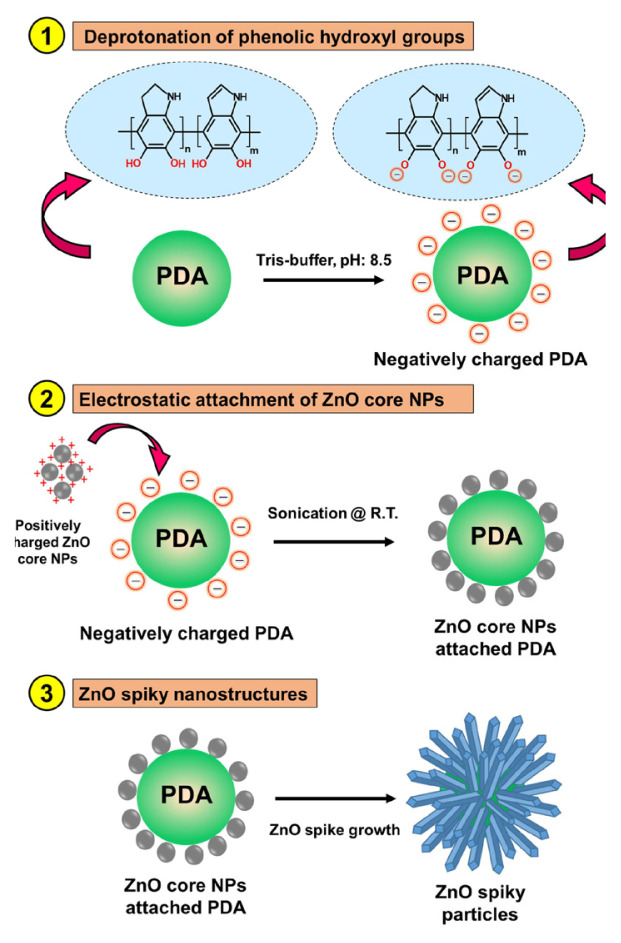
Synthesis pathway of the spiky ZnO nanostructures using PDA NPs as the starting template.

The unique dual functional properties (i.e. adhesive and hydrophilic) of PDA facilitated the development of an electroless deposition process and the growth of well-defined spherical inorganic nanostructures [13,14,17]. These distinctive characteristics drove the bottom-up assembly of spiky ZnO nanorods tailored for efficient PEC water-splitting applications. As a proof of concept for this study, a systematic seed-mediated-growth approach was adopted to fabricate spiky ZnO/Au morphologies, involving the following key steps: first, PDA NPs were synthesized via the self-polymerization of dopamine hydrochloride, which contained catechol/phenolic hydroxyl groups. These reactive functional groups exhibited zwitterionic behavior resulting from the deprotonation of phenolic groups at high pH levels (Figure 1, section 1). Consequently, the surface charge of the synthesized PDA NPs was altered from neutral to negative (Tris buffer, pH: 8.5) due to the deprotonation of phenolic hydroxyl groups. This process aided the electrostatic attachment of positively charged ZnO seed colloidal solution during ultrasonication (Figure 1, section 2). The robust ZnO nanospikes were next grown hydrothermally using a ZnO growth precursor solution containing zinc nitrate hexahydrate and hexamethylenetetramine (Figure 1, section 3). In this technique, the surface chemistry of the PDA starting templates offered dense deposition regions (i.e. catechol/phenolic hydroxyl groups) for the ZnO seed colloids, allowing seed-mediated growth of the ZnO nanospikes. Finally, the plasmonic ZnO/Au nanostructures were achieved by electrostatic interaction between the ZnO nanorods and colloidal Au NPs.

**Figure 2:**
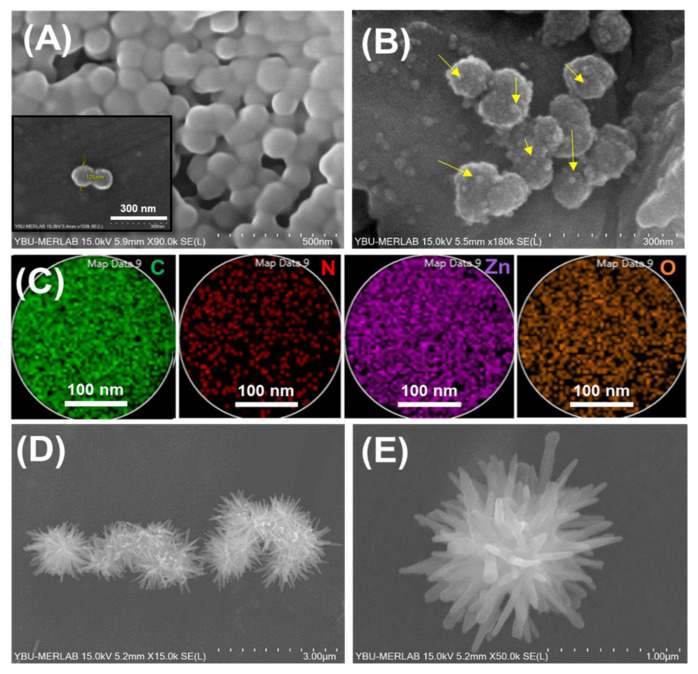
SEM images of A) bare PDA and B) ZnO seed-attached PDA NPs, C) related SEM elemental mapping of ZnO seed-attached PDA NPs, D–E) SEM images of the ZnO spiky nanostructures.

**Figure 3:**
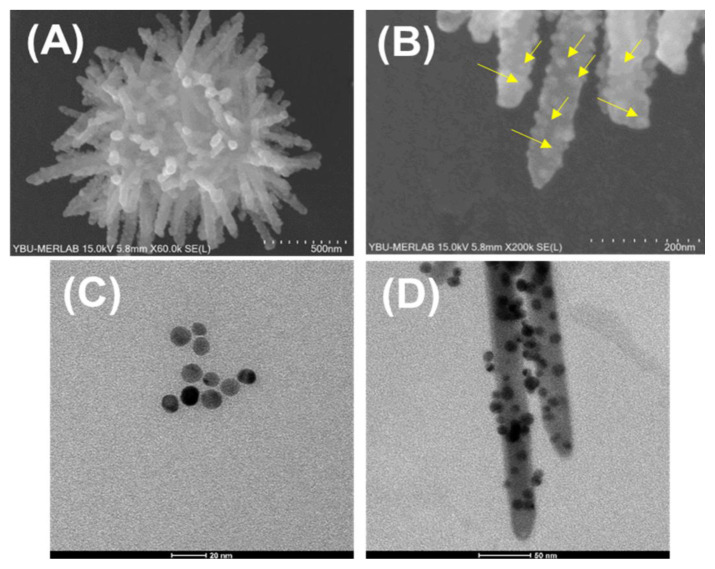
SEM images of A–B) spiky ZnO/Au nanostructures, C) TEM image of the synthesized Au NPs, and D) TEM image of the spiky ZnO/Au nanostructures.

The scanning electron microscopy-elemental mapping spectra (SEM-EDX) images of the bare PDA and ZnO seed attached NPs are represented in Figure 2. As can be seen, the PDA NPs were synthesized with a highly narrow size distribution and spherical morphology (Figure 2A). The electrostatic attachment of the ZnO seed NPs onto the PDA was achieved through the deprotonation of reactive phenol (−OH) groups with no significant changes in the PDA sphere-like shape. Note that the applied two-step synthesis pathway (PDA deprotonation and electrostatic ZnO seed attachment) ensured a dense attachment of the ZnO seed onto the PDA starting substrate due to the hydrophilicity of catechol groups (Figure 2B). In addition, the SEM-EDX elemental mappings (Figure 2C) revealed consistent distribution of C, N, Zn, and O in the ZnO-seed attached PDA NPs as well. Figures 2D and 2E, and 3A and 3B show the SEM images of the size distribution and surface morphology of the synthesized ZnO spiky structures. The ZnO spiky structures were fabricated with well-defined size distributions that retained their spherical morphology. This phenomenon resulted from the zwitterionic nature of the PDA template, which facilitated strong attachment of the ZnO seed NPs to the PDA and allowed for the production of ZnO spiky structures with the appropriate spherical morphologies.

Furthermore, the transmission electron microscopy (TEM) images of the Au NPs and Au-decorated spiky ZnO structures are shown in Figure 3. As can be seen, the Au NPs were synthesized with a narrow size distribution (average size: ~6 nm). Moreover, Figure 3C demonstrates the successful attachment of the Au onto the ZnO spiky nanostructures with no noticeable variations in the average size distribution or surface morphology of the Au-decorated spiky ZnO structures (Figure 3D).

**Figure 4:**
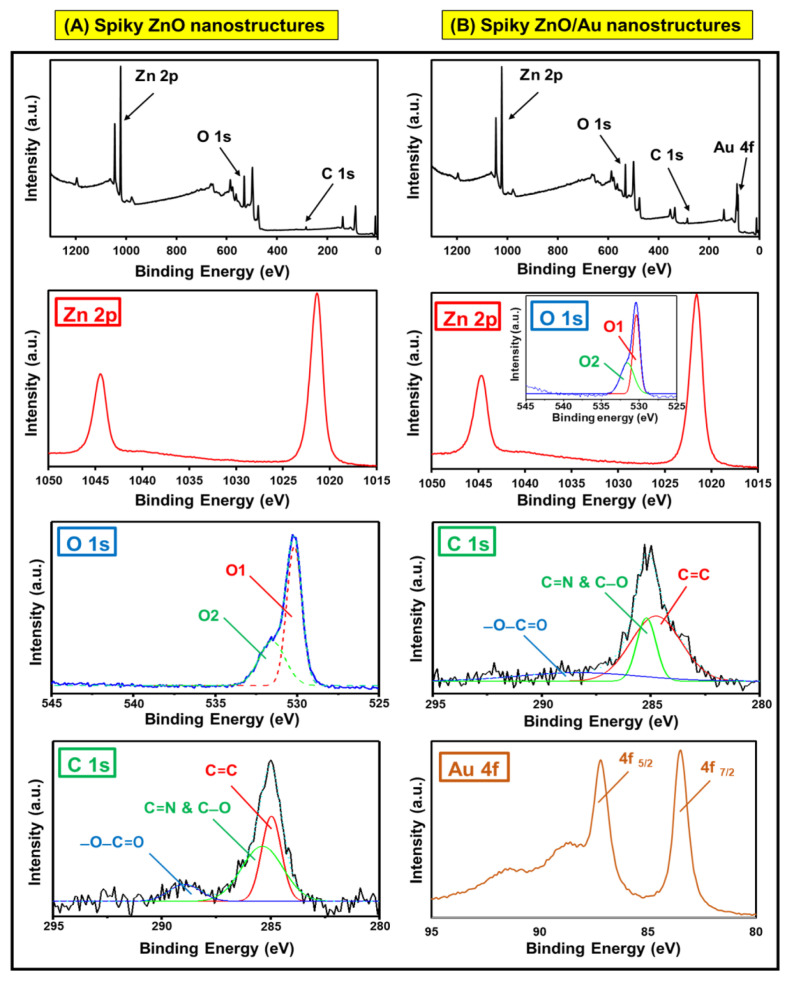
X-ray photoelectron spectroscopy survey spectra of A) the spiky ZnO nanostructures and B) the spiky ZnO/Au nanostructures.

The composition and valence states of the spiky ZnO/Au nanostructures were analyzed using XPS, revealing characteristic peaks corresponding to the Zn, O, C, and Au (Figure 4). The deconvoluted high-resolution spectra of the O 1s exhibited two peaks at binding energies of 530.3 and 532.5 eV, attributed to lattice oxygen and non-lattice (adsorbed) oxygen, respectively (Figures 4A and 4B). Additionally, the presence of the C1s core level, associated with the spin orbitals of PDA as the initial template, is shown in Figure 4A. Furthermore, the C1s core level spectra of the spiky ZnO/Au nanostructures were deconvoluted into three distinct peaks at 284.9, 287.1, and 288.4 eV, corresponding to the C=C, C=N & C-O, and −O-C=O groups, respectively (Figure 4B). Moreover, the Au 4f core level peaks (Au 4f_7/2_ at 83 eV and Au 4f_5/2_ at 87 eV) became more pronounced, confirming the presence of Au nanoparticles (Figure 4B). These well-defined Au 4f peaks confirm the metallic state of the Au nanoparticles and the successful synthesis of the intended spiky ZnO/Au nanostructures.

**Figure 5:**
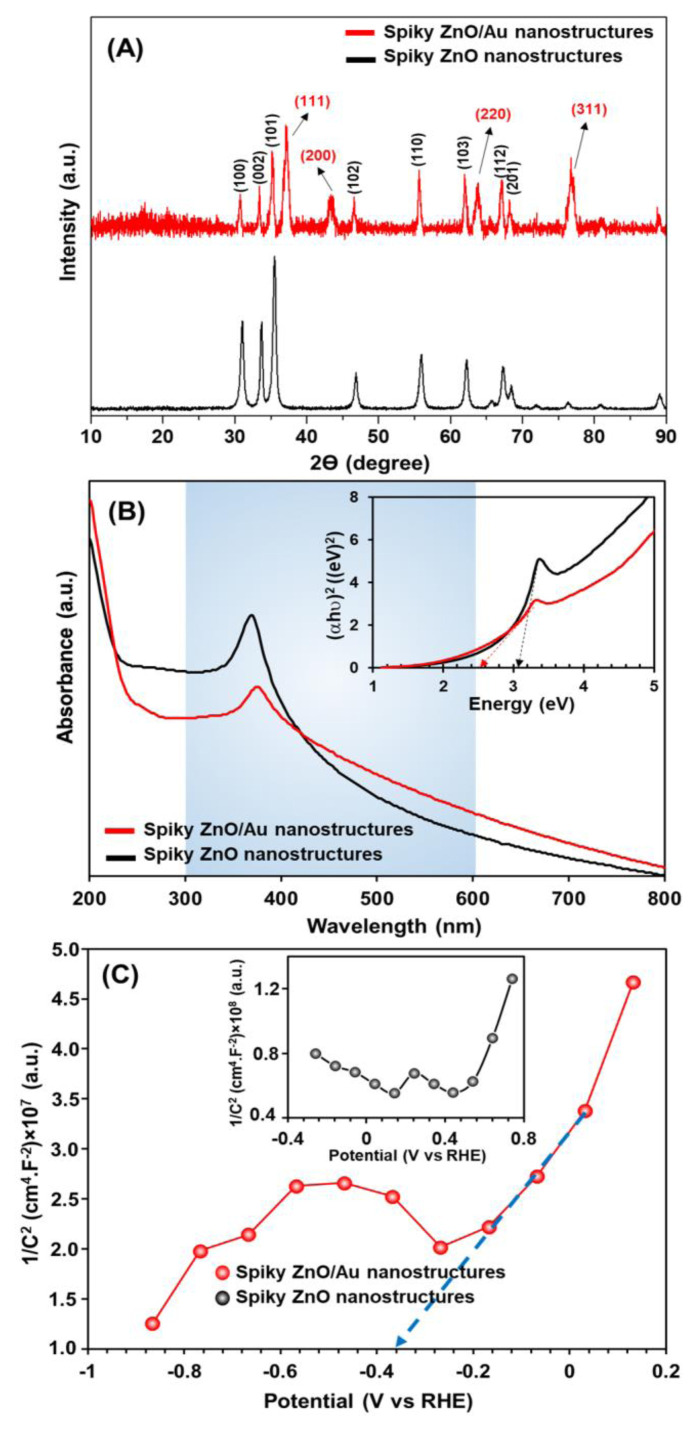
A) X-ray diffraction pattern, B) UV-vis spectra (inset: the Tauc plots), and C) Mott-Schottky of the spiky ZnO and ZnO/Au nanostructures.

The X-ray diffraction (XRD) patterns of the crystal structures of the pristine spiky ZnO and spiky ZnO/Au nanostructures are presented in Figure 5A. The XRD 2θ peaks obtained for the pristine spiky ZnO structures are marked in black (e.g., 100, 002, 101, 102, 110, 103, 112, and 201). For the spiky ZnO/Au nanostructures, the peaks corresponding to Au crystals (e.g., 111, 200, 220, and 311) are shown in red on the XRD 2θ peaks. Notably, with the attachment of Au NP, prominent peaks corresponding to Au crystals emerged alongside the peaks of the ZnO crystal form, confirming the presence of both forms distinctly. The obtained XRD patterns were in good agreement with the XPS results, which clearly demonstrated the successful synthesis of spiky ZnO/Au nanostructures (Figure 5A).

The optical characteristics and light absorption of the spiky ZnO and ZnO/Au nanostructures were studied using a UV-vis spectrophotometry (Figure 5B). The peak at ~370 nm represents the characteristic absorption of ZnO, owing to a band gap of around 3.1 eV [18]. The spiky ZnO/Au nanostructures had a shifted peak at ~380 nm and a broad band peak at ~500–600 nm, which contributed to LSPR Au NPs [18]. Note that the acquired broadband peak could be due to the good coverage of spiky ZnO nanorods by Au NPs, which increased the absorption intensity of ZnO/Au nanostructures in the visible range when compared to pristine spiky ZnO. This phenomenon revealed that the Au NPs had a superior function in boosting the performance of synthesized nanostructures when exposed to visible LED light. Furthermore, the band gap of the nanostructures was investigated using a Tauc plot, as shown in Figure 5, inset. The band gap of the nanostructures reduced from 3.1 to 2.6 eV for the pristine ZnO and spiky ZnO/Au nanostructures also, which was consistent with previous studies [18,19]. The presence of Au NPs with good LSPR capabilities resulted in active oxygen vacancies between the valance and conduction bands (CBs), altering optical absorption from UV to the visible range [17,18].

Mott-Schottky analyses were used to evaluate the conduction and valence band (VB) potentials in spiky ZnO and ZnO/Au photoanodes (Figure 5C). The flat band potentials were 0.44 and −0.36 eV for pristine ZnO and ZnO/Au, respectively. The samples had positive slopes, indicating n-type semiconductors in which the CB potential for pristine ZnO and ZnO/Au were 0.34 and −0.46 eV, respectively [10]. Using the bandgap potentials, the VB potential was 2.76 eV for pristine ZnO and 2.14 eV for ZnO/Au.

**Figure 6:**
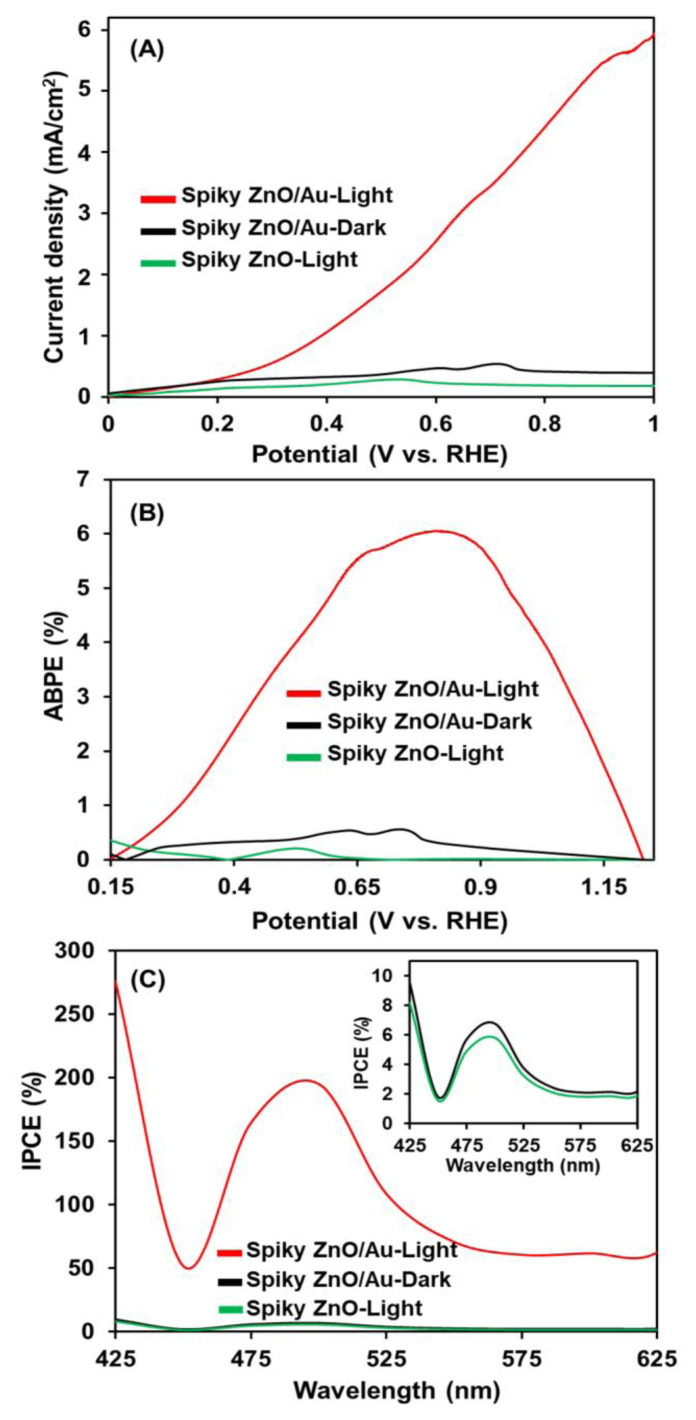
PEC water splitting performances of the spiky ZnO and spiky ZnO/Au photoanodes: A) J–V data under light and dark, B) ABPE values, and C) related IPCE spectra.

The PEC water-splitting performance of the pristine spiky ZnO and ZnO/Au photoanodes was investigated under both dark and LED light illumination conditions (30 mW/cm^2^) (Figure 6A). The spiky ZnO/Au photoanodes exhibited negligible photocurrent density under dark conditions (0.197 mA/cm^2^) due to insufficient electron-hole separation. However, under LED light irradiation, the ZnO/Au photoanodes demonstrated an improved PEC performance [5.7 mA/cm^2^ at a low potential of 1.0 V vs. the reversible hydrogen electrode (RHE)] compared to the pristine spiky ZnO photoanodes (0.017 mA/cm^2^). This enhancement in light absorption in the visible region resulted in an increased charge carrier concentration. This remarkable value was 28 times greater than that under dark conditions, attributed to effective surface plasmon resonance (SPR)-enhanced charge separation and improved hot-electron injection onto spiky ZnO nanostructures.

The PEC performance of the spiky ZnO/Au photoanodes was further evaluated using the applied bias photon-to-current efficiency (ABPE) data (Figure 6B). The highest ABPE value (6.0%) was achieved for the ZnO/Au photoanodes under LED illumination at 0.81 V vs. the RHE. Additionally, the highest ABPE results of 0.55% (at 0.74 V vs. the RHE) and 0.02% (at 0.53 V vs. the RHE) were obtained for the ZnO/Au photoanodes under dark conditions and for the pristine spiky ZnO photoanodes, respectively. These results notably demonstrate that the highest ABPE was attained due to the SPR hot-electron generation/injection, as well as efficient charge separation onto ZnO nanostructures, which enhanced the photoconversion efficiency of spiky ZnO/Au photoanodes (a 10.9-fold increase compared to dark conditions) [20]. Additionally, the incident photon-to-current conversion efficiency (IPCE) of the spiky ZnO/Au photoanodes were investigated (Figure 6C). It is evident from the data that the plasmonic photoanodes exhibited enhanced performance in the visible region (i.e. 450–575 nm) attributed to the suppression of electron-hole recombination within this spectral range. The aforementioned results highlight the significant role of effective hot-electron injection from Au to the CB of the spiky ZnO, along with the presence of abundant oxygen vacancies (both lattice and non-lattice oxygen species, Figure 4B) and enhanced light absorption in the visible region.

**Figure 7:**
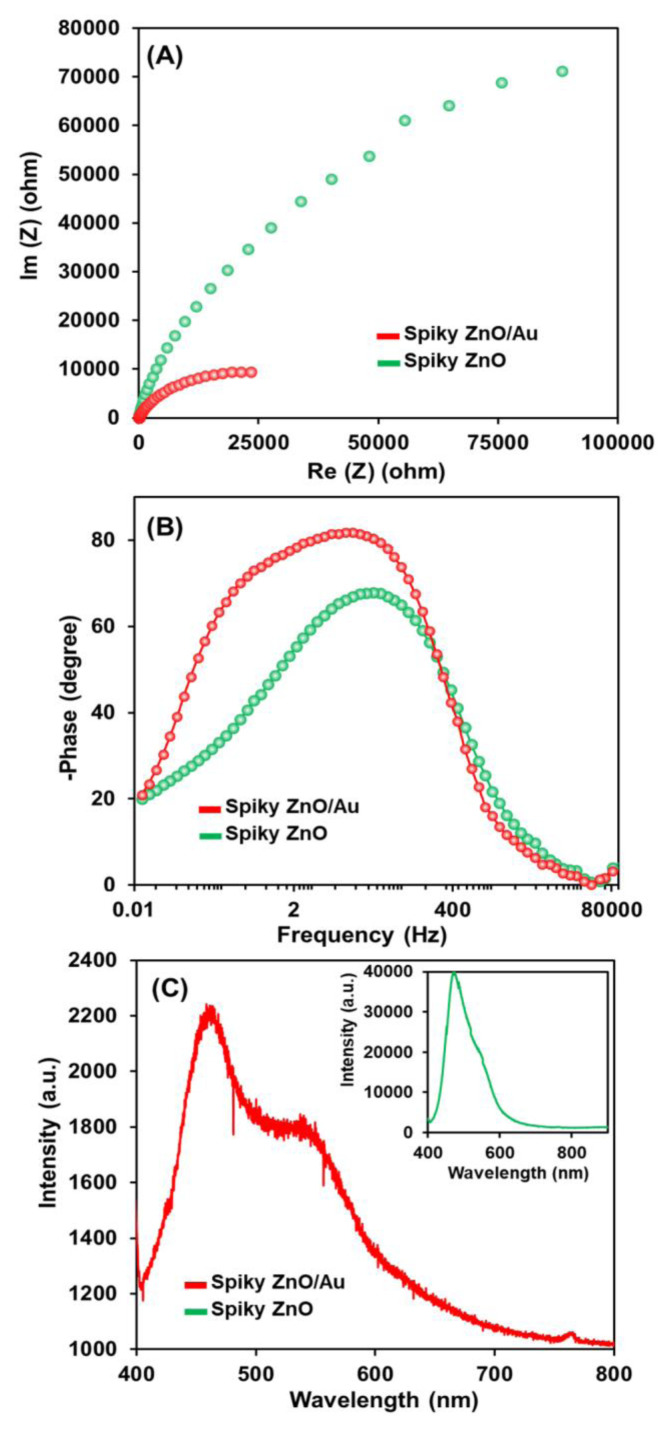
(A) Nyquist plots, (B) bode plots, and (C) PL intensity of the spiky ZnO and spiky ZnO/Au photoanodes.

The enhancement in the PEC water splitting performance of spiky ZnO/Au photoanodes can be attributed to their rapid electron transfer capability resulting from efficient electron/hole separation. This phenomenon is strongly supported by the electrochemical impedance spectroscopy (EIS) results, which indicated a smaller charge-transfer resistance for the spiky ZnO/Au photoanodes (R_ct_: 23400 Ω) compared to pristine ZnO (R_ct_: 88400 Ω) photoanodes under visible LED illumination (Figure 7A).

Furthermore, the electron lifetime in the photoanodes was assessed using Bode plots (Figure 7B), revealing that a lower peak frequency was attained for the spiky ZnO/Au photoanodes (11.37 ms). These results distinctly indicate a longer lifetime compared to the pristine ZnO photoanodes (4.5 ms), demonstrating inhibition in the electron-hole recombination. This led to an enhancement in the charge transfer efficiency and ultimately improved the photocatalytic performance for H_2_ evolution. Moreover, the efficiency of the charge separation and transfer was investigated using photoluminescence (PL), revealing a significant reduction in PL intensity for spiky ZnO/Au photoanodes, compared to pristine ZnO (Figure 7C). This reduction can be attributed to the intimate contact at the Au-ZnO interface, prolonging the lifetime of photogenerated electron-hole pairs. These findings were consistent with the EIS results, which confirmed efficient electron-hole separation and reduced electron-hole recombination under visible LED illumination.

Based on the aforementioned results and the calculated band alignments, the potential charge transfer mechanism utilizing spiky ZnO/Au photoanodes in the PEC water-splitting process is illustrated in Schemes A and B.

As depicted, under visible LED illumination, the reduction in band gap (decreased from 3.1 to 2.6 eV after Au attachment onto ZnO) enhanced the harvesting of visible light, leading to the accumulation of the photogenerated electrons on the CB of ZnO substrate Scheme B. The close interaction between the Au NPs and ZnO spikes caused a shift in the Fermi level towards the CB of ZnO, facilitating the smooth transfer of photogenerated electrons from Au to the ZnO surface. Consequently, the desired SPR-induced charge separation efficiently transferred electrons to the CB of ZnO. Since the CB of the ZnO/Au photoanodes was more negative (CB: −0.46 eV) than the redox potential of the H^+^/H_2_ (−0.41 eV), and the VB was more positive than the oxidation potential of water, the photogenerated electron-hole pairs were separated. In this scenario, the holes on Au participated in the oxidation of water, while the electrons transferred to the ZnO substrate, resulting in the reduction of water onto the Pt wire [10]. It is noteworthy that the proposed novel spiky ZnO/Au metal-dielectric substrates induced the increase of band bending at the interface, leading to enhanced visible light absorption along with efficient electron-hole separation.

## 4. Conclusion

The bottom-up assembly of spiky ZnO nanostructures with spherical shape-defined morphologies was achieved using PDA NPs. The unique dual functional groups (i.e. catechol/phenolic hydroxyl groups) along with the hydrophilic properties of PDA facilitated the development of an electroless seed-mediated growth of spiky ZnO nanorods. The well-engineered plasmonic spiky ZnO/Au metal-dielectric substrates exhibited a distinctive PEC water-splitting performance (5.7 mA/cm^2^ at a low potential of 1.0 V vs. the RHE) compared to the pristine spiky ZnO photoanodes (0.017 mA/cm^2^), owing to the enhanced light absorption in the visible region and increased charge carrier concentration. The potential charge transfer mechanism, based on the reduction in the band gap of spiky nanostructures (decreased from 3.1 to 2.6 eV after Au attachment onto ZnO), clearly demonstrated an improvement in the harvesting of visible light. This novel study proposes a low-temperature fabrication method for the construction of visible light-driven heterojunctions, with potential applications in enhanced PEC H_2_ generation, photocatalytic dye removal, supercapacitors, and biomedical fields.

## Figures and Tables

**Scheme:**
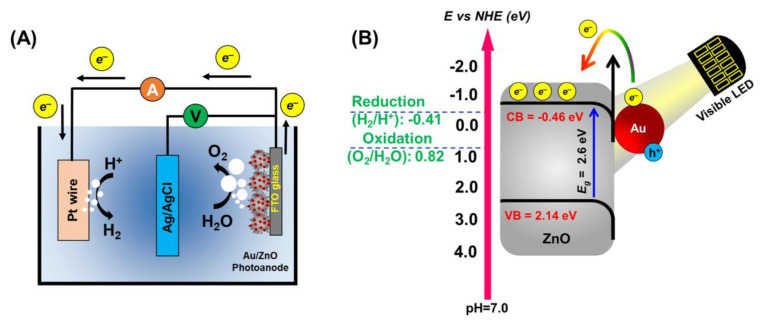
A) Schematic representation of the three-electrode PEC system, and B) the plausible charge transfer mechanism using the spiky ZnO/Au photoanodes under visible LED illumination.

## References

[1] Idrissi A Ahsaine H BaQais A Saadi M Arab M Current advances on nanostructured oxide photoelectrocatalysts for water splitting: a comprehensive review Surfaces and Interfaces 2024 45 103850 10.1016/j.surfin.2024.103850

[2] Li H Wang S Chi H Li C Photocatalytic water splitting driven by surface plasmon resonance ChemPhotoChem 2024 8 1 e202300049 10.1002/cptc.202300049

[3] Molaei MJ Recent advances in hydrogen production through photocatalytic water splitting: a review Fuel 2024 365 131159 10.1016/j.fuel.2024.131159

[4] Desai MA Sartale SD Plasmonic metal nanoparticles decorated ZnO nanostructures for photoelectrochemical (PEC) applications chemically deposited nanocrystalline metal oxide thin films: synthesis, characterizations, and applications Cham Springer International Publishing 2021 293 328 10.1007/978-3-030-68462-4

[5] Güler AC Antoš J Masař M Urbánek M Machovský M Boosting the Photoelectrochemical Performance of ZnO/Au Nanorods by Co-Occurring Gradient Doping and Surface Plasmon Modification International Journal of Molecular Sciences 2023 24 1 443 10.3390/ijms24010443 PMC9820687 36613884

[6] Wu M Chen WJ Shen YH Huang FZ Li CH In situ growth of matchlike ZnO/Au plasmonic heterostructure for enhanced photoelectrochemical water splitting ACS Applied Materials & Interfaces 2014 6 17 15052 15060 10.1021/am503044f 25144940

[7] Moon CW Choi MJ Hyun JK Jang HW Enhancing photoelectrochemical water splitting with plasmonic Au nanoparticles Nanoscale Advances 2021 3 21 5981 6006 10.1039/D1NA00500F 36133946 PMC9417564

[8] Zhang L Chen X Hao Z Chen X Li Y TiO2/Au Nanoring/p-Si nanohole photocathode for hydrogen generation ACS Applied Nano Materials 2019 2 6 3654 3661 10.1021/acsanm.9b00590

[9] Clavero C Plasmon-induced hot-electron generation at nanoparticle/metal-oxide interfaces for photovoltaic and photocatalytic devices Nature Photonics 2014 8 2 95 103 10.1038/nphoton.2013.238

[10] Abouelela MM Kawamura G Tan WK Amiruldin M Maegawa K Ag nanoparticles decorated ZnO nanopagodas for Photoelectrochemical application Electrochemistry Communications 2024 158 107645 10.1016/j.elecom.2023.107645

[11] Machín A Arango JC Fontánez K Cotto M Duconge J Biomimetic catalysts based on Au@ ZnO–graphene composites for the generation of hydrogen by water splitting Biomimetics 2020 5 3 39 10.3390/biomimetics5030039 32839383 PMC7558139

[12] Machín A Cotto M Duconge J Arango JC Morant C 2018 Hydrogen production via water splitting using different Au@ZnO catalysts under UV–vis irradiation Journal of Photochemistry and Photobiology A: Chemistry 2018 353 385 394 10.1016/j.jphotochem.2017.11.050

[13] Celebi N Aydin MY Soysal F Yıldız N Salimi K Core/shell PDA@UiO-66 metal–organic framework nanoparticles for efficient visible-light photodegradation of organic dyes ACS Applied Nano Materials 2020 3 11 11543 11554 10.1021/acsanm.0c02636

[14] Çelebi N Soysal F Salimi K Well-defined core/shell pDA@Ni-MOF heterostructures with photostable polydopamine as electron-transfer-template for efficient photoelectrochemical H2 evolution International Journal of Hydrogen Energy 2022 47 29 13828 13837 10.1016/j.ijhydene.2022.02.111

[15] Bahng JH Yeom B Wang Y Tung SO Hoff JD Anomalous dispersions of ‘hedgehog’particles Nature 2015 517 7536 596 599 10.1038/nature14092 25631447

[16] Celebi N Aydin MY Soysal F Ciftci YO Salimi K Ligand-free fabrication of Au/TiO2 nanostructures for plasmonic hot-electron-driven photocatalysis: Photoelectrochemical water splitting and organic-dye degredation Journal of Alloys and Compounds 2021 860 157908 10.1016/j.jallcom.2020.157908

[17] Salimi K Self-assembled bio-inspired Au/CeO2 nano-composites for visible white LED light irradiated photocatalysis Colloids and Surfaces A: Physicochemical and Engineering Aspects 2020 599 124908 10.1016/j.colsurfa.2020.124908

[18] Mahala C Sharma MD Basu M Near-field and far-field plasmonic effects of gold nanoparticles decorated on ZnO nanosheets for enhanced solar water splitting ACS Applied Nano Materials 2020 3 2 1153 1165 10.1021/acsanm.9b01678

[19] Perumal V Hashim U Gopinath SC Haarindraprasad R Liu WW Thickness dependent nanostructural, Morphological, Optical and impedometric analyses of zinc oxide-Gold hybrids: Nanoparticle to thin film PLoS One 2015 10 12 e0144964 10.1371/journal.pone.0144964 26694656 PMC4687870

[20] Fan K Chen H He B Yu J Cobalt polyoxometalate on N-doped carbon layer to boost photoelectrochemical water oxidation of BiVO4 Chemical Engineering Journal 2020 392 123744 10.1016/j.cej.2019.123744

